# Training-induced changes in daily energy expenditure: Methodological evaluation using wrist-worn accelerometer, heart rate monitor, and doubly labeled water technique

**DOI:** 10.1371/journal.pone.0219563

**Published:** 2019-07-10

**Authors:** Hannu Kinnunen, Keijo Häkkinen, Moritz Schumann, Laura Karavirta, Klaas R. Westerterp, Heikki Kyröläinen

**Affiliations:** 1 Optoelectronics and Measurement Techniques Research Group, University of Oulu, Oulu, Finland; 2 Biology of Physical Activity, Faculty of Sport and Health Sciences, University of Jyväskylä, Jyväskylä, Finland; 3 Department of Molecular and Cellular Sports Medicine, German Sport University, Cologne, Germany; 4 Gerontology Research Center, Faculty of Sport and Health Sciences, University of Jyväskylä, Jyväskylä, Finland; 5 Polar Electro Oy, Kempele, Finland; 6 School of Nutrition and Translational Research in Metabolism (NUTRIM), Maastricht University, Maastricht, The Netherlands; University of Thessaly, GREECE

## Abstract

**Introduction:**

Wrist-mounted motion sensors can quantify the volume and intensity of physical activities, but little is known about their long-term validity. Our aim was to validate a wrist motion sensor in estimating daily energy expenditure, including any change induced by long-term participation in endurance and strength training. Supplemental heart rate monitoring during weekly exercise was also investigated.

**Methods:**

A 13-day doubly labeled water (DLW) measurement of total energy expenditure (TEE) was performed twice in healthy male subjects: during two last weeks of a 12-week Control period (n = 15) and during two last weeks of a 12-week combined strength and aerobic Training period (n = 13). Resting energy expenditure was estimated using two equations: one with body weight and age, and another one with fat-free mass. TEE and activity induced energy expenditure (AEE) were determined from motion sensor alone, and from motions sensor combined with heart rate monitor, the latter being worn during exercise only.

**Results:**

When body weight and age were used in the calculation of resting energy expenditure, the motion sensor data alone explained 78% and 62% of the variation in TEE assessed by DLW at the end of Control and Training periods, respectively, with a bias of +1.75 (p <.001) and +1.19 MJ/day (p = .002). When exercise heart rate data was added to the model, the combined wearable device approach explained 85% and 70% of the variation in TEE assessed by DLW with a bias of +1.89 and +1.75 MJ/day (p <.001 for both). While significant increases in TEE and AEE were detected by all methods as a result of participation in regular training, motion sensor approach underestimated the change measured by DLW: +1.13±0.66 by DLW, +0.59±0.69 (p = .004) by motion sensor, and +0.98±0.70 MJ/day by combination of motion sensor and heart rate. Use of fat-free mass in the estimation of resting energy expenditure removed the biases between the wearable device estimations and the golden standard reference method of TEE and demonstrated a training-induced increase in resting energy expenditure by +0.18±0.13 MJ/day (p <.001).

**Conclusions:**

Wrist motion sensor combined with a heart rate monitor during exercise sessions, showed high agreement with the golden standard measurement of daily TEE and its change induced by participation in a long-term training protocol. The positive findings concerning the validity, especially the ability to follow-up the change associated with a lifestyle modification, can be considered significant because they partially determine the feasibility of wearable devices as quantifiers of health-related behavior.

## Introduction

Physical activity is one of the main determinants of individual energy expenditure, and the most important one to explain its changes [1]. Increasing daily total energy expenditure (TEE) supports the regulation of favorable body composition [2], and helps in the prevention and treatment of lifestyle diseases [3,4]. Various methods are commonly used to assess physical activity, including accelerometers and heart rate monitors. The most valid method to measure free-living energy expenditure, yet which is suitable for advanced research use only, is the doubly labeled water (DLW) method [5,6].

Accelerometers, that are essentially motion sensors, have become increasingly popular, both as a research tool [7] and as consumer activity trackers [8]. Wrist-worn motion sensors have been associated with higher wearing times and lower selection bias compared to waist-worn sensors [9–11]. Higher wearing times may contribute to higher validity [12] in the long term. However, the validity of wrist-worn motion sensors has been studied much less extensively than other positions—waist, chest, leg or arm—particularly in a long-term setting using DLW-assessment [5].

In a short-term measurement, using indirect calorimeter as a reference, a wrist-worn motion sensor has generally been inferior to other wearing positions, yet at its best it has shown a comparable accuracy to waist-worn sensors [13]. Only two studies have compared wrist-worn motion sensors to DLW in a long-term measurement. The first one [14] was performed with wrist-mounted GENEA accelerometers in pregnant and non-pregnant women, where a vector magnitude based linear regression model explained 26% of the variation in physical activity energy expenditure in the non-pregnant group. In the second study, an early prototype of the wrist-worn Polar Active (Polar Electro, Kempele, Finland) was used [15]; Hand motion frequency, body height and weight combined explained 74% of the variation in DLW measured TEE in soldiers. Both studies used the same population in model development and model testing, and it has not been studied how these findings can be generalized in different populations. Polar Active (Polar Electro, Kempele, Finland) is a wrist-worn motion sensor that was mainly targeted for children. Good agreement was found between Polar Active and two hip-worn actigraphy devices for the fulfillment of the daily recommendation for moderate to vigorous physical activity of 60 min in a study among 48-year old female and male subjects, even though Polar Active showed higher activity output than the hip-worn monitors [16]. Obviously, different algorithms are optimal for wrist-worn than waist-worn motion sensors.

Heart rate monitors, when individually calibrated, provide an alternative for the objective assessment of exercise induced energy expenditure [17]. According to Plasqui 2013 [5] it is not clear if other physiological data improves the validity of accelerometer methods. When combined with chest mounted motion sensor, heart rate has been shown to improve the estimation of energy expenditure in certain activities, where motion of the waist is not closely related to energy expenditure [18]. Importantly, chest-worn motion sensor combined with heart rate monitor has been shown to increase the accuracy over motion sensor in a long-term DLW-assessment [19] as well. Continuous wearing of a chest-worn heart rate monitor over several days can be uncomfortable. Subsequently, the latest wearable products have incorporated optical measurement of heart rate into wrist-worn devices providing reasonable accuracy in several types of physical activities [20].

DLW is considered the golden standard for long-term assessment of daily energy expenditure. Overall validity of activity monitors can be judged with DLW-assessed activity induced energy expenditure (AEE) as a reference [1]. Observation for 1 or 2 weeks of free-living activity is likely to include the full range of physical activity behavior of an individual [2]. DLW does not separate the contribution of different intensities to TEE. High cost level, special instrumentation and the technical expertise that is required have limited the application of DLW in research. A benefit of motion sensor based assessment of physical activity is the ability to track the amount and timing of activities at different intensity zones, such as inactivity, light-, moderate- and vigorous intensity.

The present study focuses on the daily energy expenditure and its’ long-term changes and utilizes DLW as the reference method. In short-term measurements, indirect calorimetry has been widely used as the reference method for assessing the validity of accelerometers in various sports and non-sports activities [21]. Energy metabolism is estimated from respiratory gas exchange measurements utilizing ventilation volume, O_2_ and CO_2_ concentrations in exhaled air so that O_2_ consumption and CO_2_ production can be measured and transformed into estimates of energy expenditure via metabolic equations. It has achieved the golden standard status in the estimation of acute energy metabolism due to high accuracy and reproducibility [22]. Indirect calorimeter is the only method that can provide reliable calibration data in free-living conditions across different intensities from resting levels to high intensity exercise. However, it is not suitable for long-term measurement.

Aerobic and strength training have different characteristics in respect to their effects on human energy expenditure during exercise, between exercise sessions, and in the long-term training (Table 1). Aerobic exercise significantly increases energy expenditure during and a few hours after cessation of exercise: the excess post-exercise consumption accounts only 6–15% of the acute net effect [23]. On the other hand, energy expenditure during strength exercise is usually at a moderate level while it can increase REE for several days after a single bout of exercise [24]. Long-term participation in strength training also induces an increase in REE via increased lean body mass; Lean body mass explains up to 80% of the between subject variability in REE [25]. Subsequently, with motion sensor and heart rate monitor, it is quite straightforward to assess the increased energy expenditure of dynamic aerobic training. However, it is more challenging to assess the corresponding effect associated with strength training: acceleration sensor does not reflect static workload during strength exercises nor any changes in REE following exercise. The ability of heart rate monitor to quantify excess post exercise energy expenditure is also limited because heart rate response after exercise varies for many reasons between individuals [26].

**Table 1:** Contribution of sedentary life, aerobic and strength training to daily energy expenditure during exercise, between exercise sessions, and after long-term participation in training.

Activity type (Level*)	During 45min exercise ^#^	Between exercise sessions	Long-term effects
Inactive life (1.5 MET)	0.4 MJ	-	Decline in fitness, increase of fat mass, decrements in global body functioning further contribute to an inactive lifestyle and decreased daily EE [27].
Strength training, such as moderate to vigorous circuit training (4.3–8.0 MET)	1.1–2.0 MJ	Resting EE mildly elevated for days, such as +0.2–0.4 MJ/day for 3 days in [6]. The post-exercise effect of intense bouts can be significant, and in the case of short, intense exercises, it may even exceed the acute effect.	Increases lean body mass and elevates resting EE, preserves functional capacity [28].
Aerobic training, such as running 8–11 km/h (8.3–11.0 MET)	2.1–2.8 MJ	Resting EE elevated for 3–12 hours, totals 6–15% of the acute effect [9].	Increases aerobic fitness and promotes active lifestyle. May decrease other habitual daily physical activity, e.g. in older adults in [29].

EE: Energy Expenditure.

*) Example physical activity level estimated in metabolic equivalents (MET) according to [21] (codes 07022, 02035, 02040, 12030, and 12070).

^#)^ the corresponding acute accumulated EE for a 45-min exercise session for a subject with basal metabolic rate 8.0 MJ/day (a value typical for a 30-year old male person who weighs about 80 kg).

Wearable devices and mobile applications provide real time behavioral feedback to users which may increase common understanding of healthy activity levels. This target may not be reached unless the accuracy and reliability are good enough [30]. To be embedded for continuous use in wearable devices, the data processing and algorithms should not include an excess amount of computing or memory use. There is a subsequent need for robust methods to estimate the intensity of physical activity and its role in daily energy expenditure.

The aims of the present study were: 1) To assess the validity of a wrist-worn motion sensor in estimating daily energy expenditure in non-athletic male subjects in their normal physical activity state, and after long-term lifestyle change of added participation in regular aerobic and resistance exercise. 2) To evaluate whether the combination of motion sensor and heart rate monitor improves the estimation of daily energy expenditure over the motion sensor alone, considering that the training program consisted of stationary aerobic and resistance exercises. 3) To assess the ability of the combination of motion sensor and heart rate monitor to detect the change in TEE and AEE induced by the lifestyle change of added physical training.

There are no prior references on the DLW-assessed validity of wrist-worn motion sensor in non-athletic male population. The special novelty of the present study is the evaluation of wrist-worn motion sensor in detecting a change in energy expenditure induced by a lifestyle modification. This paper also introduces a robust method for combining continuous motion sensor and intermittent heart rate data into a minute-by-minute estimate of the intensity of physical activity.

## Materials and methods

### Subjects

Fifteen males participated in the present study (Table 2). Subject selection was based on preferential randomization among a group of participants (n = 21) in a larger study that evaluated combined strength and aerobic training. All subjects of the larger study received detailed requirements of the sub-study and were asked to provide a written notice of interest to participate in the sub-study. Out of these subjects, the total number of 15 was then randomly drawn. The larger study has been described earlier in detail [31]. Briefly, prior to inclusion into the study, the subjects were moderately physically active as characterized by irregular participation of walking, cycling or occasionally team sports for not more than 3 times per week. No-one systematically engaged in any endurance or strength training before inclusion into the study. Exclusion criteria included pronounced overweight (BMI ≥ 30 kg/m^2^) as well as acute and chronic illness or diseases that would contraindicate intense physical exercise. A completed health questionnaire and resting ECG were reviewed by a cardiologist prior to commencement of the study.

**Table 2:** Descriptive statistics of the subjects, and their adherence to wearing the motion sensor.

Parameter	Control	Training	Change	P
Body mass (kg)	81.6±12.1	82.1±11.8	0.43±1.49	.148
BMI (kg/m^2^)	25.1±3.4	25.2±3.3	0.14±0.47	.153
FFM (kg)	58.0±6.5	60.0±7.2	1.97±1.48	**<.001**
FM (kg)	23.5±6.6	22.3±6.0	-1.26±1.24	**.002**
Fat%	28.4±4.6	26.7±4.5	-1.68±1.15	**<.001**
${\dot{\text{V}}\text{O}}_{2\text{peak}}(\text{ml}/\text{kg}/\text{min})$	35.7±7.2	38.7±4.7	2.9±3.7	**.009**
1RM Leg Press (kg) ^a)^	144.4±25.6	157.0±22.9	12.6±4.1	**<.001**
Wearing time (%)	93.2±14.1	87.3±19.8	-5.8±12.7	.213
Wearing or night (%)	98.4±2.7	97.4±4.0	-1.0±2.9	.125

Values are mean±SD; n = 13; P-value indicates significance of the Change (increase/decrease). BMI: Body Mass Index; FFM: Fat Free Mass, FM: Fat Mass, ${\dot{\text{V}}\text{O}}_{2\text{peak}}$: peak oxygen consumption tested on treadmill. 1RM: one repetition maximum. Wear time is percentage of the duration of the 13-day DLW-assessment.

^a)^ One subject did not attend the final maximal strength test, hence n = 12.

The study was conducted according to the Declaration of Helsinki and ethical approval was given by the Ethics Committee at the University of Jyväskylä. Information about the possible risks of all study procedures were provided both verbally and in writing before subjects gave their written informed consent.

### Study timeline and training protocol

The subjects underwent a 12-week Control period followed by supervised training of 12 weeks (Training). During Control period, the subjects were asked to maintain their activities of daily living, but no prescribed physical exercise was conducted. During the training of 12 weeks, 4 weekly sessions of strength and aerobic (2 x strength and 2 x aerobic) exercise sessions were conducted on alternating days.

The strength training program consisted of heavy resistance loading focusing on muscle growth and maximal strength development (mainly 2–5 sets for 8–10 repetitions at 80–85% of 1RM and 2–5 sets with 3–5 repetitions at 85–95% of 1RM) including typical exercises (6–8 exercises per session) for the lower and upper extremities and trunk. Exercises for the lower body consisted of bilateral dynamic leg press, as well as both bilateral (weeks 1–7) and unilateral (weeks 8–12) dynamic knee extension and flexion. Additional exercises for the upper body included vertical shoulder press and lateral pull down, as well as exercises commonly used to improve trunk stability. The overall duration of each strength protocol was 30–50 min. During weeks 1–2, all exercises were conducted with a circuit model using 2–4 sets of 15–20 repetitions at an intensity of 40–60% of 1RM. During the following 10 weeks of training, protocols aiming for muscle hypertrophy (2–5 × 8–10 repetitions at 80–85% of 1RM, 1.5–2 min rest between the sets) and maximal strength (2–5 × 3–5 repetitions at 85–95% of 1RM, 3–4 min rest between sets), as well as during the last 2-week period protocols targeting explosive strength (2 × 8–10 repetitions at 40% of 1RM, 3–4 min rest between the sets) were incorporated into the training program.

The aerobic training program included both steady-state and interval exercise sessions on a bicycle ergometer. The training intensity was controlled by the heart rate monitor. The intensity was progressively increased from steady-state cycling below the individually determined aerobic threshold during weeks 1–7 and included interval sessions above the lactate threshold during weeks 8–12. Similarly, the duration of endurance cycling progressively increased throughout the 12 weeks of training from 30 to 50 minutes. Subjects were instructed to maintain a constant pedaling frequency at approximately 70 rpm during each exercise session, while the magnetic resistance of the ergometer was adjusted to achieve the required heart rate response. The duration of cycling within each training session was 30–50 min.

The validation of the wrist worn motion sensor by the DLW method was performed over 2-week periods during the final weeks of the Control and Training periods. All 15 selected subjects completed the Control period, however 2 subjects dropped out during the Training period due to personal reasons.

### $\dot{\text{V}}\text{O}2\text{peak}$ and muscle strength measurement protocols

Peak oxygen consumption (${\dot{\text{V}}\text{O}}_{2\text{peak}}$) was determined at the start and at the end of the 12-week training period via an incremental cycling test on a bicycle ergometer (Ergometrics 800, Ergoline, Bitz, Germany). The protocol begun at 50 W and increased by 25 W every 2 minutes. Subjects were asked to maintain a pedaling frequency of 70 rpm throughout the test. The test was stopped when the subjects failed to maintain the required cadence for more than 15 seconds. Oxygen uptake was determined breath-by-breath using a gas analyzer (Oxycon Pro, Jaeger, Hoechberg, Germany). On each testing day, air flow calibration was performed using a manual flow calibrator. Before each test, automatic air flow calibration was performed, and the gas analyzer was calibrated using a certified gas mixture of 16% O_2_ and 4% CO_2_. ${\dot{\text{V}}\text{O}}_{2\text{peak}}$ was calculated as the highest ${\dot{\text{V}}\text{O}}_{\text{2}}$ value averaged over 60 seconds, and HR_max_ was the average HR during the last 60 seconds of the test.

One repetition maximum (1RM) of leg extensors was determined at the start and at the end of the 12-week training period using a dynamic horizontal bilateral leg press device (David 210, David Health Solutions, Helsinki, Finland). Following a warm up, a maximum of 5 trials were allowed to obtain a true 1RM. The greatest load that the subject could lift to full knee extension at an accuracy of 1.25 kg was accepted as 1RM. One subject was unable to perform the leg press test at the end of the training period.

### Energy expenditure and body composition assessed by doubly labeled water

DLW based determination of average daily TEE and Total Body Water were measured according to the Maastricht protocol [32]. Briefly, following the collection of a baseline urine sample (day 0), the subjects ingested a weighed amount of ^2^H_2_^18^O, resulting in an initial excess body water enrichment of 150 ppm for deuterium and 300 ppm for oxygen-18. Subsequent urine samples were collected in the morning of days 1, 8 and 14 and in the evening of days 1, 8, and 13 of the DLW measurement periods. Body composition parameters (fat-free mass and fat mass) were calculated based on the 0.73 ratio of total body water to fat-free mass [33].

Resting energy expenditure (REE) was calculated using both the Wang [34] and Schofield [35] equations. The selected Wang equation utilizes fat-free mass (REE_FFM_ (MJ/day) = 0.0908 · FFM(kg) + 1.565), and the selected Schofield equation utilizes body weight and age (REE_BW_, Eqs 1 and 2). The FFM based estimation was selected because it also adjusts to body composition change that can be expected as a response to long-term participation in strength training. Additionally, the body weight and age-based estimation was selected since required data in more easily accessible and the corresponding equations are more widely used in wearable devices.

$${REE}_{BW}\left(\frac{MJ}{day}\right)=0.0632\cdot BW(kg)+2.895,if\;age\;18-30\;years$$ (1)

$${REE}_{BW}\left(\frac{MJ}{day}\right)=0.0481\cdot BW(kg)+3.653,if\;age\;18-30\;years$$ (2)

Activity induced energy expenditure (AEE) was calculated as 0.9 x TEE–REE; the equation assumes the thermic effect of nutrients to be 10% of TEE, which is based on a normal mixed diet [2].

### Energy expenditure assessed by wrist motion sensor and heart rate monitor

The subjects were instructed to wear a motion sensor (Polar Active, Polar Electro, Kempele, Finland) on their wrist throughout the 24-week study protocol, and a heart rate monitor (Polar RS800CX) during both supervised exercise sessions and any voluntary physical activity when they expected that the intensity exceeded that of walking. The subjects were also instructed to wear the heart rate monitor when biking. In this study, the motion sensor and heart rate monitor outputs were selected over the two-week periods corresponding with the timing of the DLW assessments.

The motion sensor included a capacitive 1-D accelerometer (VTI Technologies, Vantaa, Finland; currently Murata Electronics). The measurement principle of the motion sensor has been presented earlier [15]. Briefly, hand motion frequency and regularity are determined for every 30 seconds, and they are transformed to the Physical Activity Level (in MET units) via an adjustment for body height, with higher body height corresponding to higher activity level at similar motion frequency. The motion sensor units selected for this investigation were tested for consistency before the study period to avoid dropouts due to technical problems.

#### Combining motion sensor data with heart rate data into a single estimate of energy expenditure

The Physical Activity Level (in MET units) time series, which are stored in 30 second epochs by the motion sensor, were exported from the Polar gofit.com web service for further analysis. TEE derived from motion sensor was calculated by multiplying the Physical Activity Level with both estimates of REE. TEE derived from motion sensor and exercise heart rate data was calculated by replacing the motion sensor derived energy expenditure with the corresponding estimation from heart rate monitor whenever the latter was available and indicated moderate to vigorous physical activity (> 4 MET). 4 MET corresponds with walking 5 km/h, and our guidance for subjects was to wear a heart rate monitor during voluntary training when they expected the intensity to exceed that of walking. The heart rate monitor derives the energy expenditure estimation using individually measured ${\dot{\text{V}}\text{O}}_{2\text{peak}}$ and maximal heart rate [17,36]. It was visually verified that each subject had worn the heart rate monitor during each supervised exercise session without obvious measurement errors.

#### Detection and substitution of non-wear time data during night and day

**Fig 1:**
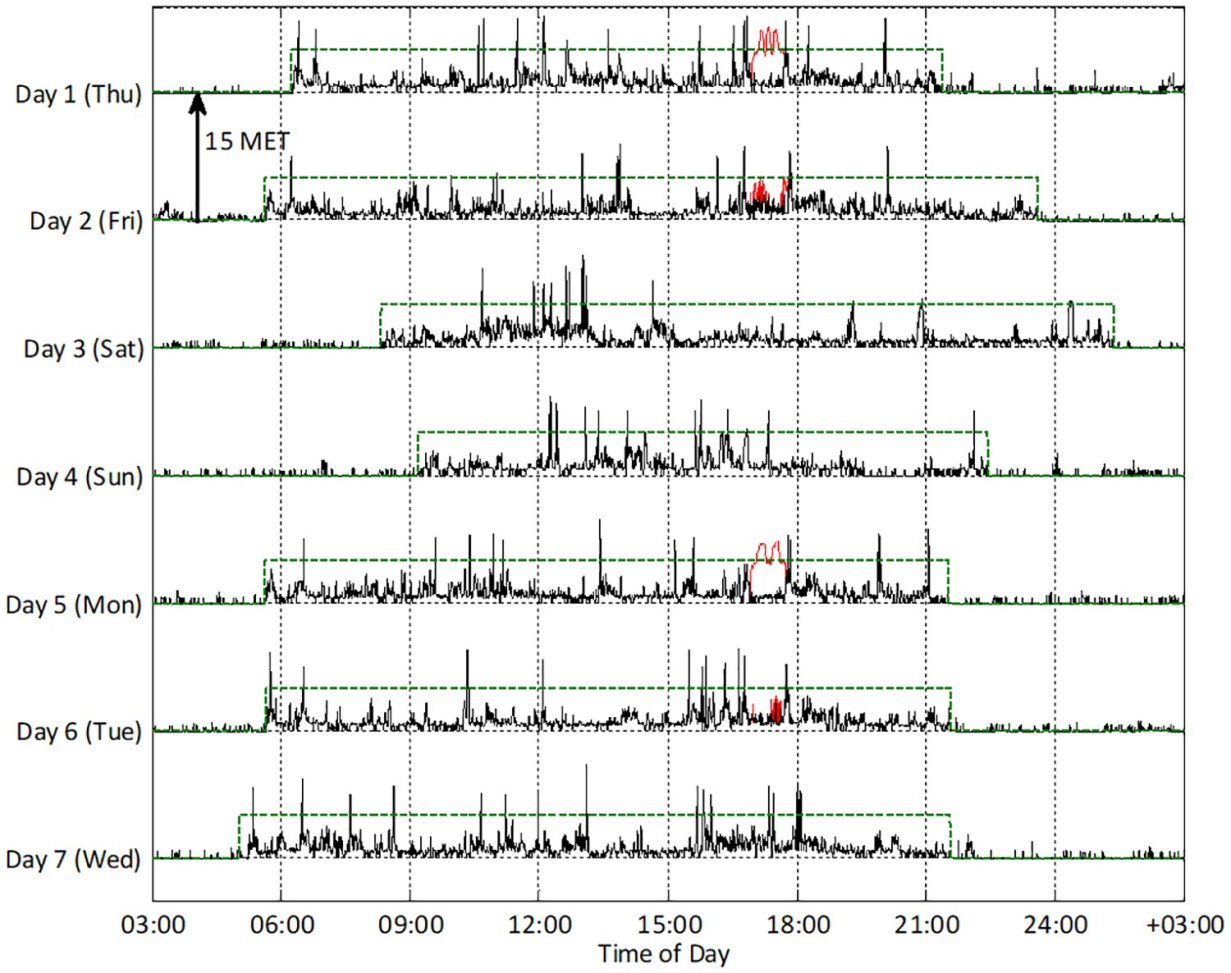
Seven days of physical activity intensity measured by the wearable sensors from one subject. Solid black line shows data measured by the motion sensor 24/7, and solid red line is data from heart rate monitor during four weekly bicycle ergometer and strength exercise sessions. Dashed green line marks time-out-of-bed and time-in-bed.

Initially, time in bed was estimated from MET data if the majority of 30 second epochs in a moving 10-minute window indicated full rest–Polar Active records 0.875 MET as the sleeping metabolic level after 10 minutes of full rest. Final daily time-to-bed and time-out-of-bed were determined visually as shown in Fig 1. Data corresponding to time in bed were set to the sleeping metabolic level. Non-wear was detected as a consequent absence of movements for more than 90 minutes, a time window that is comparable to that used previously [37]. If non-wear was detected in connection to night-time (between 11.30pm and 11am), the physical activity level was set to the sleeping metabolic level for the corresponding period of time. Occasional daily non-wear data (Table 2) was replaced with individual average physical activity level of daily wear time.

### Calculated training-induced change in TEE

To support statistical power analysis, we calculated the change in TEE that could be expected as a result of our training program. Since training-induced energy expenditure is only a minor part of TEE, and participant behavior may change outside the exercise sessions over the course of the 24-week study, this calculation was targeted to provide a reference value at the group level only. The expected metabolic requirements of aerobic and strength exercise sessions were calculated based on Compendium of Physical Activities [21] with the assumption that exercise [4 x strength (6.0 MET) and 4 x aerobic exercise sessions (8.0 MET) within 13 days, 50 min per session] would replace sedentary (1.5 MET) behavior. To keep this approximation simple, REE was estimated from body weight for each subject.

### Nutritional intake

Subjects were instructed to maintain normal dietary intake throughout the 24-week study period. To quantify energy intake, the subjects were requested to complete food diaries during the first consecutive three days, and last consecutive three days of each 2-week measurement period. To assure approximately similar energy intake during both measurement periods, food diaries of the first three days during the measurement period 1 were returned to the subjects in the beginning of the second measurement period.

### Distribution of intensity of physical activity

To quantify the effect of participation in the training program on habitual daily activity, the distribution of daily time according to the intensity of physical activity was calculated: inactive (1–2 MET), light (2–4 MET), moderate (4–7 MET) and vigorous intensities (>7MET). One-minute moving window with 30 seconds overlapping was used. 4 and 7 MET cut-points were selected to match to the corresponding values used in the motion sensing product that was used in this study (Polar Active).

### Statistical analysis

Data are presented as mean ± SD. The significance of the changes induced by the training intervention were assessed with non-parametric signed Wilcox rank sum test. Because all comparisons were taken within an individual, we used a paired sign rank test, and because we were testing specifically for uni-directional change, i.e. increase from the Control period to the Training period, we applied the one-tail test specifying increase. This was executed in Matlab (MathWorks Inc, MA, USA) using the signrank function. Similarly, the equivalence of the change in TEE and AEE detected by the wearable devices, and the reference method DLW, were assessed with a paired sign rank test applying one-tailed test specifying an increase or a decrease. Results were deemed statistically significant when p < 0.05.

For assessment of validity of the wearable devices in determining TEE and AEE, linear regression analysis using Spearman correlation was performed between the outputs from the wearable devices and the reference method DLW. Bland-Altman analysis [(Wearable–DLW) vs. DLW] was used for the assessment of mean and proportional bias between the methods; a positive bias indicates that the wearable devices overestimate daily energy expenditure outcomes, while a negative bias indicates an underestimate. 95% Limits of Agreement (LoA) were processed from a mean difference ± 1.96 · SD. Data processing was performed using the Matlab software.

Due to the high cost of the DLW assessment, our budget allowed inclusion of 15 subjects. We calculated our statistical power in respect to the ability of motion sensor and heart rate monitor to detect an increase in TEE from moderately active life to regular training 4 times weekly. The null hypothesis was that TEE during the Training period is similar to the Control period. The alternative hypothesis was that TEE during the Training period is higher than during the Control period. For the statistical power analysis, inactive life for the young male subjects was estimated to represent a physical activity level of 1.5 x REE, which leads to an estimated TEE of 12 MJ/day in our 20-40-year old male population. The calculated training-induced change was +1.1 MJ/day. Between subject standard deviation was estimated to be 1.5 MJ/day, i.e. somewhat lower than in our past study in highly active soldiers [15]. Based on the above assumptions, with our N = 15, the statistical power to reject the null hypothesis with 5% type I error rate was 88%. Maximum 3 dropouts were to be allowed for the statistical power to stay above 80%.

## Results

### Descriptive statistics and training-induced changes

The age of the study participants was 30±6 years and body height was 1.80±0.08 m. Additional subject characteristics are presented in Table 2.

Over the 12-week training program the attendance rate in prescribed endurance and strength exercise sessions was 100%. ${\dot{\text{V}}\text{O}}_{2\text{peak}}$ increased during the training period by 2.9±3.7 ml/kg/min (p = .009) equaling to 10.0±11.3%. 1RM Leg Press results increased by 12.6±4.1 kg (p <.001) corresponding to 9.3±4.8%. Fat-free mass increased by 1.97±1.48 kg (p <.001) and fat mass decreased by 1.26±1.24kg (p = .002). The average wear-time of motion sensor was 93±13% and 87±20% during the 2-week DLW assessments in the Control and Training conditions, respectively. Most of the non-wear occurred overnight, since day-time wear and night-time together accounted for 98±3% and 97±4% during Control and Training conditions, respectively.

### Daily energy expenditure during control and training periods

**Fig 2:**
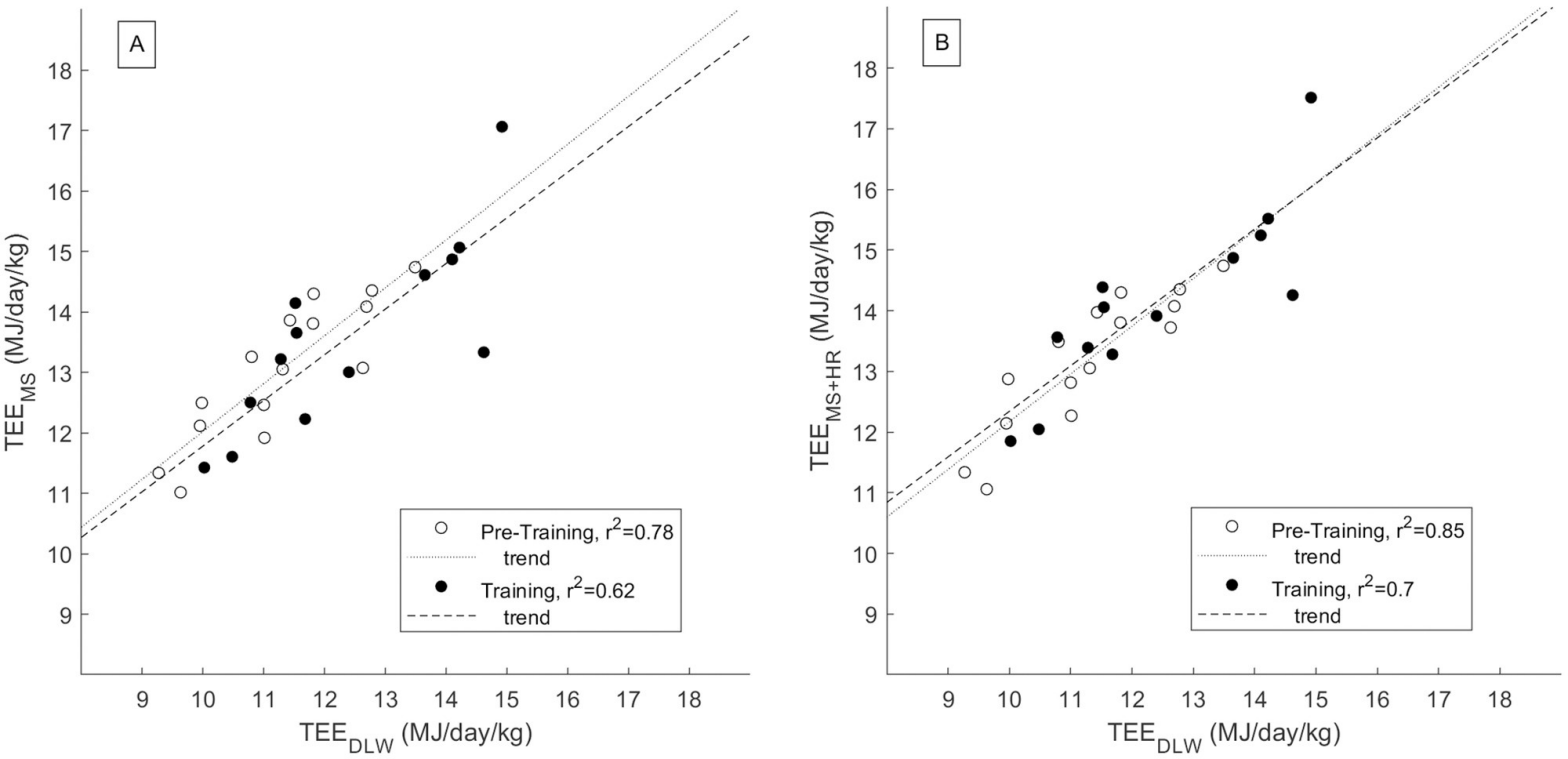
Wearable sensors data explain significant portion of the between-subject changes in DLW-assessed total energy expenditure. (A) Regression plot for DLW and Motion Sensor assessed Total Energy Expenditure (TEE_DLW_ and TEE_MS_). Open dots from the Control period (N = 15), closed dots from the Training period (N = 13). (B) Corresponding plot when the motion sensor data is replaced by heart rate monitor data during exercise sessions (TEE_MS+HRM_). In A and B, resting energy expenditure was estimated using body weight and age [35].

When REE was calculated from body weight, the motion sensor approach explained 78% and 62% of the variation in TEE assessed by the DLW during the Control and Training periods, respectively, with a bias of +1.75 (p <.001) and +1.19 MJ/day (p = .002). Correspondingly, the combination of motion sensor and exercise heart rate explained 85% and 70% of the variation with bias of +1.89 and +1.75 MJ/day (p <.001 for both). Fig 2 shows the scatter plot between these variables. When REE was calculated from fat free mass, the motion sensor approach explained 72% and 62% of the variation in TEE assessed by the DLW during the Control and Training periods, and combination of motion sensor and exercise heart rate explained 72% and 65%, without any statistically significant biases. Table 3 demonstrates the results from the Linear Regression analysis and Bland-Altman analysis in more detail for both TEE and AEE. Bland-Altman analysis did not reveal proportional biases between the methods.

**Table 3:** Regression and Bland-Altman agreement analysis of daily energy expenditure outputs of doubly labeled water and the wearable sensors. Results presented for the Control and Training periods.

Comparison	r^2^	Bias (sig.)	95% LoA	trend (sig.)
***Control period***	*REE using body weight and age*			
TEE_MS_ vs. TEE_DLW_ (MJ/day)	0.78	1.75 (**p <.001**)	0.53;2.98	-0.23 (p = .419)
TEE_MS+HRM_ vs. TEE_DLW_ (MJ/day)	0.85	1.89 (**p <.001**)	0.77; 3.02	-0.40 (p = .137)
AEE_MS_ vs. AEE_DLW_ (kJ/kg/day)	0.58	19.9 (**p <.001**)	4.2; 35.6	0.04 (p = .893)
AEE_MS+HRM_ vs. AEE_DLW_ (kJ/kg/day)	0.60	21.6 (**p <.001**)	6.1;37.0	-0.08 (p = .793)
	*REE using fat free mass*			
TEE_MS_ vs. TEE_DLW_ (MJ/day)	0.72	0.16 (p = .314)	-1.21; 1.53	-0.13 (p = .648)
TEE_MS+HRM_ vs. TEE_DLW_ (MJ/day)	0.72	0.28 (p = .121)	-1.00; 1.56	-0.12 (p = .667)
AEE_MS_ vs. AEE_DLW_ (kJ/kg/day)	0.54	1.9 (p = .296)	-14.4; 18.2	0.09 (p = .753)
AEE_MS+HRM_ vs. AEE_DLW_ (kJ/kg/day)	0.54	3.4 (p = .126)	-12.3; 19.1	-0.09 (p = .743)
***Training period***	*REE using body weight and age*			
TEE_MS_ vs. TEE_DLW_ (MJ/day)	0.62	1.19 (**p = .002**)	-0.76; 3.15	-0.05 (p = .863)
TEE_MS+HRM_ vs. TEE_DLW_ (MJ/day)	0.70	1.75 (**p <.001**)	0.03; 3.46	-0.38 (p = .202)
AEE_MS_ vs. AEE_DLW_ (kJ/kg/day)	0.43	13.1 (**p = .002**)	-9.2;35.4	-0.03 (p = .921)
AEE_MS+HRM_ vs. AEE_DLW_ (kJ/kg/day)	0.50	19.3 (**p <.001**)	-1.5;40.2	-0.08 (p = .807)
	*REE using fat free mass*			
TEE_MS_ vs. TEE_DLW_ (MJ/day)	0.62	-0.24 (p = .277)	-2.21; 1.74	0.10 (p = .751)
TEE_MS+HRM_ vs. TEE_DLW_ (MJ/day)	0.65	0.25 (p = .105)	-1.48; 1.98	-0.02 (p = .949)
AEE_MS_ vs. AEE_DLW_ (kJ/kg/day)	0.43	-2.6 (p = .271)	-24.8; 19.5	-0.10 (p = .751)
AEE_MS+HRM_ vs. AEE_DLW_ (kJ/kg/day)	0.53	2.9 (p = .108)	-17.0; 22.8	-0.05 (p = .863)

n = 15 during Control and n = 13 during Training period. LoA: Limits of Agreement in BA-plot; trend (sig.): slope and its significance in BA-plot indicating proportional bias between the methods. TEE: Total Energy Expenditure. AEE: Activity Induced Energy Expenditure. DLW: assessed by doubly-labeled water. MS: assessed by Motion Sensor (24/7); MS+HR: assessed by MS (24/7) and Heart Rate Monitor (during exercise sessions).

### Training-induced changes in TEE, AEE, and REE

Based on the duration and intensity of the supervised exercise sessions, the calculated change in TEE was +1.08±0.10 MJ/day. Table 4 summarizes training-induced changes in TEE, AEE, and REE. All methods detected a significant increase in TEE and AEE. A significant increase in REE was observed when fat-free mass based calculation was applied (p <.001). When body weight based calculation of REE was applied, comparison to the DLW assessed change showed that the change in TEE estimated by the motion sensor approach was underestimated (+1.13±0.66 vs. 0.59±0.69, p = .004 by DLW and motion sensor, respectively) while the estimated change using combined wearable sensor approach did not differ from that of the golden standard DLW method. Compared to the golden standard measure of the change in AEE, the motion sensor approach underestimated the change (11.9±6.1 kJ/kg/day vs. 5.7±7.3 kJ/kg/day, p = .005, by DLW and motion sensor, respectively) while the corresponding change obtained by the combined wearable sensor approach did not differ from the golden standard DLW measurement. When fat-free mass based estimation of REE was applied, the estimated change in total daily energy expenditure from motion sensor and the combined wearable sensor approaches were not statistically different from the corresponding DLW measurement. In comparison to the change in AEE measured by the DLW, the motion sensor underestimated the change (10.0±6.4 kJ/kg/day vs 6.0±6.3 kJ/kg/day, p = .034, by DLW and motion sensor, respectively) while the corresponding result from the combination of motion sensor and exercise heart rate measurement was not different from the measured change in AEE by the golden standard DLW.

**Table 4:** Training-induced change in daily energy expenditure (Mean±SD); comparison between control and training periods (n = 13).

Parameter	Control	Training	Change	P
	*REE using body weight and age*			
REE_BW_ (MJ/day)	7.82±0.56	7.85±0.54	0.03±0.09	.133
TEE_DLW_ (MJ/day)	11.27±1.35	12.40±1.69	1.13±0.66	**<.001**
TEE_MS_ (MJ/day)	13.00±1.22	13.60±1.57	0.59±0.69	**<.001**
TEE_MS+HRM_ (MJ/day)	13.17±1.19	14.15±1.48	0.98±0.70	**<.001**
AEE_DLW_ (kJ/kg/day)	28.6±11.2	40.5±14.5	11.9±6.1	**<.001**
AEE_MS_ (kJ/kg/day)	48.3±11.7	54.0±14.1	5.7±7.3	**.002**
AEE_MS+HRM_ (kJ/kg/day)	50.2±12.2	60.3±14.2	10.1±7.2	**<.001**
	*REE using fat free mass*			
REE_FFM_ (MJ/day)	6.83±0.59	7.01±0.65	0.18±0.13	**<.001**
TEE_DLW_ (MJ/day)	11.27±1.35	12.40±1.69	1.13±0.66	**<.001**
TEE_MS_ (MJ/day)	11.37±1.29	12.16±1.66	0.79±0.57	**<.001**
TEE_MS+HRM_ (MJ/day)	11.51±1.27	12.65±1.60	1.14±0.58	**<.001**
AEE_DLW_ (kJ/kg/day)	40.9±10.8	50.9±14.2	10.0±6.4	**<.001**
AEE_MS_ (kJ/kg/day)	42.3±11.0	48.3±13.2	6.0±6.3	**.001**
AEE_MS+HRM_ (kJ/kg/day)	43.9±11.5	53.9±13.2	9.9±6.1	**<.001**

Change from Control to Training period. p-value indicates the significance of the change. REE_BW_: Resting Energy Expenditure estimated from Body Weight and Age. TEE_DLW_: Total Energy Expenditure assessed by DLW. TEE_MS_: TEE assessed by Motion Sensor. TEE_MS+HRM_: TEE assessed by Motion Sensor and Heart Rate Monitor. AEE: Activity Induced Energy Expenditure. REE_FFM_: Resting Energy Expenditure estimated from Fat Free Mass.

S1 Table illustrate individual day-to-day energy expenditure data determined from wearable devices data and calculated REE. Data are presented separately for control days, non-exercise days and exercise days. Wearable devices showed higher energy expenditure on exercise days than non-exercise days. Correspondingly, S2 Table summarizes daily intake values derived from food diaries. The results indicate a general inter-individual association between measured energy expenditure and reported food intake, while food intake is clearly under-reported by the study subjects–roughly by 20%. Reported food intake was similar irrespective of daily exercise program.

### Training-induced changes in physical activity at different intensities

Table 5 shows daily physical activity across intensity zones, and the change observed when participating in the training intervention. During the Training period, motion sensor detected an increase in moderate and vigorous physical activity of 12±8 (p <.001) and 3±3 min/day (p <.001), respectively. MS+HR detected an increase in moderate and vigorous physical activity of 24±8 (p <.001) and 7±5 min/day (p <.001). Further on, the combination of motion sensor and exercise heart rate measurement indicated a tendency towards decreased inactive time of -38±70 min/day (p = .064).

**Table 5:** Daily accumulated time (h:mm, mean±SD) at different intensities of physical activity as detected by wearable devices during the control and training periods, and their corresponding change.

Parameter	Control	Training	Change	p
	*Motion Sensor only (24/7)*			
Nightly rest time	8:48±0:49	8:40±0:49	-0:08±0:26	0.175
Inactive 1–2 MET	9:01±1:23	8:32±1:54	-0:29±1:12	0.121
Light 2–4 MET	5:23±1:28	5:44±1:35	+0:22±0:58	0.200
Moderate 4–7 MET	0:43±0:14	0:55±0:19	+0:12±0:08	**<.001**
Vigorous >7 MET	0:05±0:05	0:08±0:05	+0:03±0:03	**<.001**
	*Motion Sensor (24/7) + Heart Rate Monitor during exercise*			
Nightly rest time	8:48±0:49	8:40±0:49	-0:03±0:34	0.202
Inactive 1–2 MET	9:00±1:23	8:22±1:51	-0:38±1:10	0.064
Light 2–4 MET	5:17±1:29	5:32±1:39	+0:09±0:56	0.433
Moderate 4–7 MET	0:46±0:14	1:09±0:20	+0:24±0:08	< **.001**
Vigorous >7 MET	0:09+0:07	0:16±0:07	+0:07±0:05	**<.001**

p-value indicates significance of the change from Control to Training periods. Motion sensor: Polar Active. Heart rate monitor: RS800CX. A moving window with 1 min window length during daytime, and 10 min during nightly rest was applied.

## Discussion

Data derived from two wearable devices—a wrist-worn motion sensor worn 24 hours a day throughout the study and a heart rate monitor worn during exercise sessions—explained significant portions of the variation of TEE_DLW_ (85 and 70%) during moderately active life and when participating in a training intervention. The training intervention induced several favorable health and physical fitness related changes, including improved ${\dot{\text{V}}\text{O}}_{2\text{peak}}$, improved maximal muscle strength, and decreased fat mass. The intervention also resulted in increased daily physical activity, and a remarkable increase in DLW-assessed daily energy expenditure. The combination of the two wearable devices enabled detecting the increase in TEE.

Wrist mounted motion sensor alone explained 78% of the variation of TEE_DLW_ during the Control and 62% during the Training period, respectively. These values are well in line with the best results from the corresponding studies where an accelerometer has been placed on the waist or chest [5]. The motion sensor used in the present study derives the MET levels from hand motion frequency and body height [6,15,38,39]. This method is different from the traditional vector magnitude count or a more recently proposed mean amplitude deviation method [14,16]. Using the motion frequency based method, our group earlier demonstrated high agreement with DLWassessment in soldiers [15], yet in that study the motion frequency based model was fine-tuned for the same sample of subjects. In the present study, no sample-specific adjustment was performed; instead, commercially available consumer devices and their embedded algorithms were used. Our findings about the validity of the motion frequency-based approach indicates that at least with wrist-worn motion sensors, motion frequency may represent whole body movement better than the traditional vector magnitude based features. Reliability in the assessment of daily energy expenditure, and ability to follow-up a change associated with a lifestyle change, partially determine the feasibility of wearable devices as objective quantifiers of health-related behavior. Daily TEE can also be a marker of health. At best, wearable devices may provide a mapping tool for individual, and population scale health behavior.

Among devices that have been validated against DLW for the estimation of daily EE, the SenseWear armband (BodyMedia, Inc., Pittsburgh, PA) can be considered to be the closest alternative for wrist-mounted motion sensor–however, the device is no longer available, and it included more parameters in the estimation (skin temperature, galvanic skin response and heat flux). Also wider limits of agreement have been reported than in the present study [40,41]. It must also be noted that new consumer grade devices have been developed over the recent years [20,41] but validation against DLW is not available.

The change in daily TEE measured by the golden standard DLW method (+1.13±0.66 MJ/day) as a result of the present training program was well in line with the expected change (+1.08±0.10 MJ/day), yet the measured change included a notable variability between individuals. Together with potential changes in non-exercise physical activity during the 24-week assessment, the measurement error of DLW can also account for some variability. However, when compared to TEE assessed by a respiration chamber, high general agreement has been observed between DLW and the chamber, e.g. 0±6% (mean±SD) [42]. A change in individual TEE resulting from a modest lifestyle modification, such as starting to exercise once or twice weekly, may not always exceed the measurement error limits of the DLW assessment. However, in the present study the average change in the total daily energy expenditure assessed by DLW was 10.0%, which clearly exceeds the expected error limits.

The intensity distribution of daily physical activities, as detected by motion sensor and combined motion sensor and exercise heart rate approaches, demonstrated an increase in moderate to vigorous physical activity. Participation in the exercise intervention was reflected as average increases of moderate to vigorous physical activity by 15 and 31 min/day for MS and MS+HR, respectively. Theoretically, the present training program (8 sessions in 13 days, 50 min/session) induced an additional 34 minutes of moderate to vigorous intensity activity daily. The observed relationship of increased physical activity between the methods was consistent with the corresponding relationship in estimated change in TEE. Both methods indicated significant increases in TEE and AEE during the training intervention. However, the motion sensor approach underestimated the changes, and a more accurate estimate was determined when the combination of motion sensor and exercise heart rate was used. By combining the accumulated time in moderate to vigorous physical activities and change in TEE and AEE, it can be confirmed that the underestimate of motion sensor can be attributed to the inability of motion sensors to properly detect the intensity of bicycle ergometer and strength workouts, and an inability to detect the long-term metabolic effects of strength training that occurred via favorable changes in body composition.

The present 24-week study demonstrated wide individual variability in the training-induced changes in time spent at different intensity zones, indicating that some individuals may have also changed their non-exercise physical activity behavior during the Training period. Earlier studies have even indicated a systematic compensatory reduction in non-exercise physical activity in response to aerobic and resistance exercise programs [43], which was not the case in this study. Non-exercise physical activity may also contribute to improved fitness and other positive outcomes that are generally attributed to training [38]. Also in an earlier study, the motion sensor approach was shown to be a feasible method for quantifying the expected levels of non-exercise physical activity outside actual exercise sessions [39].

A significant 14–17% overestimation was observed in TEE estimated using the motion sensor data and exercise heart rate. The motion sensor (Polar Active) has been developed for children. Body dimensions, cardiovascular fitness and movement patterns change from childhood to adulthood, and these factors at least partially explain why equations created for children overestimated the energy expenditure for adult male population. Adjustments are clearly needed when Polar Active is used in adults. The most straightforward way would be to lower the MET levels relative to the amount and intensity of movement detected. The observed ~15% overestimation could be compensated by lowering MET values for body heights that are typical for adults (~3.5 MET vs. 4 MET) in the model that estimate intensity of physical activity at a given motion rate for a given body height. The observed overestimation and subsequent need for a downward adjustment, are in line with earlier findings [16] where Polar Active indicated higher MET values than hip-mounted sensors. In addition, since REE represents majority of daily TEE and since sleeping metabolic level was already set to a low value (0.875 MET) in Polar Active, an ideal solution would also include a REE estimation that takes body composition—or related parameters—more strongly into account. The widely used REE estimation equations that utilize body dimensions, age and gender as proxies for body composition, were developed decades ago. The relation between these factors and body composition has changed over the recent decades [25].

TEE assessed by DLW includes resting metabolism, but it cannot be quantified separately. Subsequently, we also derived AEE from DLW assessed total energy expenditure by adjusting for estimated REE, the largest component of TEE. We acknowledge that lack of individual REE assessment by indirect calorimetry (respiratory gas analysis) is a limitation in the present study. However, REE was estimated from lean body mass as measured with isotope dilution. Thus, training effects on REE were detected trough changes in lean body mass, the main determinant of REE [25]. It was shown that the use of fat-free mass, instead of body weight and age in the estimation of REE, removed significant biases in the estimation of mean daily TEE and AEE. Long-term positive training adaptations include improved body composition, as was observed in this study. The increase in REE that arose from increased fat-free mass, accounted for +0.18 MJ/day that equates to 16% increase in TEE induced by the 12-week training period. These findings support the use of body composition in the estimation of REE. However, the measurement or estimation of body composition requires special analysis methods and skilled personnel, and it may not be easily applicable in consumer wearable devices. In research settings, bioimpedance measurement might provide the most feasible tool to provide better estimates for REE in the future. Even though the use of fat-free mass in the calculation of REE removed the biases between the wearable devices and DLW when estimating TEE and AEE, it did not narrow the limits of agreement with respect to DLW.

The main limitation of the present study is its relatively small sample size (n = 13). However, due to high validity of the DLW analyses and very low number of DLW validations in wrist-worn devices it is reasonable to run validation studies even with relatively small samples. Another limitation is that the intensity of a strength training cannot be controlled and analyzed using heart rate like during endurance exercise, since strength training intensity is essentially defined by neuromuscular rather than cardiovascular load. A typical endurance training session consisted of intensities slightly below the aerobic threshold for a duration of 30–40 minutes and included bouts at and above the lactate threshold. Finally, all subjects in this study were healthy young adult males, which warrants additional studies in different populations. Validating a new method and technology using data collected from a different population from the original development population can reveal certain limitations. The present devices did not include complex modeling; instead, they consisted of piecewise linear estimates of intensity of PA as a function of hand motion frequency [15], and that of HR [17]. The robustness of the algorithms may support their generalizability in different populations—yet the systematic overestimation observed in this study demonstrates a need for a systematic adjustment.

### Conclusions

As a result of participating in the 12-week combined aerobic and strength training intervention, an increased total energy expenditure was observed together with favorable changes in cardiovascular fitness, maximal muscle strength and body composition among moderately active, healthy men. Daily energy expenditure estimates derived via a wrist-mounted motion sensor was strongly associated with corresponding two-week DLW-assessment both during a Control period, and when participating in regular training. As hypothesized, improved agreement with DLW-assessed total and activity induced energy expenditure was achieved by complementing motion sensor with a heart rate monitor during weekly exercise sessions. A combination of wrist motion sensor (Polar Active) and heart rate measurement during exercise (Polar RS800CX) allowed a valid estimate of the change in daily total and activity induced energy expenditure resulting from participation in a training program. While the present results revealed a significant overestimation in the studied wearable device outcomes, positive findings that confirm their validity, especially their ability to detect a change associated with a lifestyle modification, can be considered significant because this helps determine the feasibility of wearable devices as quantifiers of health-related behavior.

## Supporting information

S1 Table Individual daily energy expenditure from Wearables. Data are averaged separately for control days, non-exercise days and exercise days.
(PDF)

S2 Table Reported daily intake on exercise and non-exercise days. Data from control period, and separately on non-exercise and exercise days from training period.
(PDF)

S1 Dataset Data necessary to replicate the present study findings. (XLSX)
