# Supplementary material for: Training-induced changes in daily energy expenditure: Methodological evaluation using wrist-worn accelerometer, heart rate monitor, and doubly labeled water technique
Source: PLoS One. 2019 Jul 10;14(7):e0219563. doi: 10.1371/journal.pone.0219563 (PMC6619827; doi:10.1371/journal.pone.0219563)
Supplement: S1 Table — Data are averaged separately for control days, non-exercise days and exercise days. (PDF) [file pone.0219563.s001.pdf]

S1 Table. Individual daily energy expenditure based on wearable devices data and calculated REE. Data are averaged separately for control days, non-exercise days and exercise days.

	**No Training**	**Regular Training**	
**Subject**	CNTRL (13 days) [MJ/d]	EXE (8 days) [MJ/d]	NonEXE (5 days) [MJ/d]
1	10.57	12.43	10.99
2	9.47	10.97	9.82
3	9.41	10.62	9.37
4	12.62	14.88	12.33
5	10.51	11.83	10.80
6	11.47	12.35	11.46
7	11.05	13.64	10.10
8	12.41	14.00	13.11
9	12.80	16.68	14.33
10	13.50	15.99	13.84
11	12.52	14.22	11.92
12	11.08	13.52	11.08
13	12.41	14.78	13.76
Mean	*11.53*	*13.53*	*11.76*

REE estimated from Fat-free mass using Wang equation [33].

CNTRL = normal day when not participating in training, EXE = exercise day when participating in training (endurance and strength exercise), Non-EXE = non-exercise day between exercise days.
