# Supplementary material for: Training-induced changes in daily energy expenditure: Methodological evaluation using wrist-worn accelerometer, heart rate monitor, and doubly labeled water technique
Source: PLoS One. 2019 Jul 10;14(7):e0219563. doi: 10.1371/journal.pone.0219563 (PMC6619827; doi:10.1371/journal.pone.0219563)
Supplement: S2 Table — Data from control period, and separately on non-exercise and exercise days from training period. (PDF) [file pone.0219563.s002.pdf]

S2 Table. Reported Daily Intake of control days, exercise and non-exercise days. Data are averaged for control days, non-exercise days and exercise days.

	**No Training**		**Regular Training**			
	CNTRL		EXE		NonEXE	
Subject	[MJ/d]	[days]	[MJ/d]	[days]	[MJ/d]	[days]
1	8.07	6	7.30	4	8.46	2
2	7.99	6	8.73	4	6.77	2
3	6.89	6	6.94	4	6.12	2
4	10.70	6	10.17	4	10.10	3
5	6.13	6	7.62	4	8.74	2
6	8.97	6	8.89	4	7.71	2
7	9.43	6	10.45	4	9.45	2
8	10.09	6	10.42	4	11.18	2
9	11.34	6	11.14	4	11.07	2
10	11.39	8	7.50	4	11.49	2
11	10.06	8	10.60	4	11.72	2
12	10.25	8	11.79	3	6.80	3
13	9.40	8	9.99	3	10.40	3
Mean	*9.29*	*6.4*	*9.35*	*4.0*	*9.23*	*2.1*

CNTRL = normal day when not participating in training, EXE = exercise day when participating in training (endurance and strength exercise), Non-EXE = non-exercise day between exercise days.
